# Optimization of [^18^F]PSMA-1007 PET-CT using regularized reconstruction in patients with prostate cancer

**DOI:** 10.1186/s40658-020-00298-8

**Published:** 2020-05-12

**Authors:** Elin Trägårdh, David Minarik, Gustav Brolin, Ulrika Bitzén, Berit Olsson, Jenny Oddstig

**Affiliations:** 1 Clinical Physiology and Nuclear Medicine, Skåne University Hospital and Lund University, Carl Bertil Laurells gata 9, 205 02 Malmö, Sweden; 2 Wallenberg Centre for Molecular Medicine, Lund University, Lund, Sweden; 3 Medical Radiation Physics, Skåne University and Lund University, Malmö, Sweden; 4 Medical Radiation Physics, Skåne University Hospital and Lund University, Lund, Sweden; 5 Clinical Physiology and Nuclear Medicine, Skåne University and Lund University, Lund, Sweden

**Keywords:** PET-CT, PSMA, Block-sequential regularized expectation maximization, Q.Clear, Image quality, [18F]PSMA-1007

## Abstract

**Background:**

Prostate-specific membrane antigen (PSMA) radiotracers such as [^18^F]PSMA-1007 used with positron emission tomography-computed tomography (PET-CT) is promising for initial staging and detection of recurrent disease in prostate cancer patients. The block-sequential regularization expectation maximization algorithm (BSREM) is a new PET reconstruction algorithm, which provides higher image contrast while also reducing noise. The aim of the present study was to evaluate the influence of different acquisition times and different noise-suppressing factors in BSREM (*β* values) in [^18^F]PSMA-1007 PET-CT regarding quantitative data as well as a visual image quality assessment.

We included 35 patients referred for clinical [^18^F]PSMA-1007 PET-CT. Four megabecquerels per kilogramme were administered and imaging was performed after 120 min. Eighty-four image series per patient were created with combinations of acquisition times of 1–4 min/bed position and *β* values of 300–1400. The noise level in normal tissue and the contrast-to-noise ratio (CNR) of pathological uptakes versus the local background were calculated. Image quality was assessed by experienced nuclear medicine physicians.

**Results:**

The noise level in the liver, spleen, and muscle was higher for low *β* values and low acquisition times (written as activity time products (ATs = administered activity × acquisition time)) and was minimized at maximum AT (16 MBq/kg min) and maximum *β* (1400). There was only a small decrease above AT 10. The median CNR increased slowly with AT from approximately 6 to 12 and was substantially lower at AT 4 and higher at AT 14–16. At AT 4–6, many images were regarded as being of unacceptable quality. For AT 8, *β* values of 700–900 were considered of acceptable quality.

**Conclusions:**

An AT of 8 (for example as in our study, 4 MB/kg with an acquisition time of 2 min) with a *β* value of 700 performs well regarding noise level, CNR, and visual image quality assessment.

## Background

Prostate cancer remains one of the most common malignancies affecting men worldwide [1, 2]. Positron emission tomography-computed tomography (PET-CT) is a widely accepted imaging technique for initial staging and for detecting sites of disease recurrence in patients with prostate cancer; this has particularly been the case since the introduction of prostate-specific membrane antigen (PSMA) radiotracers [3]. PSMA has emerged as one of the most favourable targets for PET imaging, as it is a transmembrane protein that is highly overexpressed in prostate cancer cells. The most commonly used PSMA tracer in PET imaging is [^68^Ga]Ga-PSMA-11 but there is a growing interest in ^18^F-labelled PSMA agents, which offer advantages with respect to image resolution and production amount. One of the promising ^18^F-labelled PSMA radiotracers is [^18^F]PSMA-1007 [4].

A novel generation of PET scanners based on silicon (Si)-photomultiplier (PM) technology that was introduced to the market a few years ago can potentially improve pathology detection, due primarily to its higher sensitivity [5–7]. Concurrently, improved reconstruction algorithms such as the block-sequential regularization expectation maximization algorithm (BSREM) [8, 9], commercially known as Q.Clear (GE Healthcare, Milwaukee, WI, USA) [10], have been described. Compared to ordered subset expectation maximization (OSEM), BSREM can maintain a low noise level as the number of iterations increases. This reduces the need of “stopping early” as a strategy for noise control and allow for more iterations while keeping the noise level at clinical acceptable levels. The BSREM algorithm suppresses noise via a penalty factor *β*, in which higher values not only suppress noise more but also reduce resolution. Lesion detectability has been found to be equal or higher with BSREM as compared to OSEM [11, 12]. BSREM have also been shown to increase the quantitative accuracy of standardized uptake value (SUV) measurements, particularly in small lesions [9]..

To the best of our knowledge, the impact of the *β* factor, acquisition time, and administered activity for image quality has never been investigated for [^18^F]PSMA-1007. The aim of the present study was to evaluate the influence of different activity time products (ATs = administered activity × acquisition time; in our study we have kept administered activity fixed) and different *β* values (range 300–1400) on quantitative data as well as visually assessed image quality in [^18^F]PSMA-1007 SiPM-based PET-CT. A secondary aim was to find the lowest possible AT with adequate image quality.

## Methods

### PET-CT system

Four Discovery MI (GE Healthcare, Milwaukee, WI, USA) PET-CT systems were used for image acquisition at the Department of Clinical Physiology and Nuclear Medicine, Skåne University Hospital, Lund or Malmö, Sweden. The PET-CT systems were equipped with time-of-flight and configured with four rings of detector blocks with lutetium yttrium oxyorthosilicate crystals coupled to an array of SiPM. The crystal size was 4.0 × 5.3 × 25 mm^3^ and the PET detector had a transaxial field of view of 70 cm, an axial field of view of 20 cm, and an overlap of 24% between multi-bed positions (per vendor recommendation). The sensitivity was 13 cps/kBq according to NEMA standards. The PET-CT systems were cross-calibrated to a dose calibrator; the calibration is validated monthly using an SUV control with phantoms. The PET systems were combined with a 128-slice CT.

### Patients and imaging

We included 35 patients aged 18 years or older, referred for clinical [^18^F]PSMA-1007 PET-CT due to initial staging (*n* = 14, all high-risk prostate cancer based on clinical T stage, prostate-specific antigen level and/or Gleason score) or biochemical recurrence (*n* = 21, mean prostate-specific antigen 3.6, range 0.2–16) of prostate cancer, between 16 September and 7 October 2019. The mean (± standard deviation, SD) weight was 93 ± 15 kg (range 62–124 kg), the mean BMI was 29 ± 4 (range 21–38), and the mean age was 70 ± 7 years (range 52–78 years).

Imaging was performed 120 min after radiotracer administration, and the patients were asked to empty the bladder before the image acquisition. The patients were scanned from the mid-thigh to the base of the skull. The mean administered [^18^F]PSMA-1007 activity was 4.0 ± 0.1 MBq/kg (range 4.3–3.8), while the mean accumulation time was 121 ± 3 min (range 115–129 min). The PET images were reconstructed using the commercially available BSREM algorithm Q.Clear (GE Healthcare, Milwaukee, WI, USA) including the time-of-flight and point spread function modelling with a 256 × 256 matrix (pixel size 2.7 × 2.7 mm^2^, slice thickness 2.8 mm). Images were acquired for 4 min/bed position in list mode. Sinograms with acquisition times of 1.0, 1.5, 2, 2.5, 3, 3.5, and 4 min/bed were created from the list files. These were reconstructed with 12 different *β* values, 300–1400 (increments of 100), thus yielding 84 image series per patient.

Administered activity and acquisition times are, to a close approximation, interchangeable so long as the count rate is within the linear part of the noise-equivalent count rate curve, which can be assumed in the case of clinical [^18^F]PSMA studies: 2 MBq/kg with an acquisition time of 4 min/bed is equivalent to 4 MBq/kg and 2 min/bed, assuming the same accumulation time between administration and scan time (2 h in this study). Therefore, going forward, we will use AT to refer to the product of the administered activity per unit of body weight and the acquisition time (MBq/kg × min/bed), to emphasize that the results do not depend on the acquisition time alone but, rather, on the combination of time and administered activity. However, it should be remembered that in this study, we used a fixed administered activity of 4 MBq/kg.

CT images were acquired for attenuation correction of the PET images and for anatomic correlation. A diagnostic CT with intravenous and oral contrast was performed. The tube current modulation was applied by adjusting the tube current for each individual with a noise index of 37.5 and a tube voltage of 100 kV. The slice thickness was 0.625 mm. The CT used for attenuation correction was acquired in the late venous phase. The adaptive statistical iterative reconstruction technique was applied.

This study was approved by the Regional Ethical Review Board (#2016/417) and was performed in accordance with the Declaration of Helsinki. All patients provided written informed consent.

### Image analysis

Images were evaluated quantitatively (image noise, contrast-to-noise ratio (CNR), and SUVmax) and qualitatively (visual image assessment), and both the quantitative and qualitative analysis were taken into account for the subsequent overall recommendation on what AT and *β* value to choose.

### Quantitative analysis

Two image quality metrics were used for quantitative analysis of the reconstructed PET images: image noise in normal tissue (liver, spleen, and muscle) and the CNR of pathological uptake sites versus the local background. In addition, the SUV in the liver, spleen, and muscle was calculated for all patients at AT 16 and *β* 500. Two patients were excluded from the quantitative analysis due to a lack of pathological uptakes.

The image noise level was calculated from regions of interest (ROIs) in the liver, spleen, and muscle, drawn on transaxial images using Hermes 2.0.0 (Hermes Medical Solutions, Stockholm, Sweden). Three 5-cm^2^ ROIs were drawn in transaxial slices with one slice in between; these measurements were averaged. None of the ROIs were placed where liver metastases or large vessels were seen. The ROIs were drawn in the 4 min image series and copied to the other series. The noise level was quantified as the coefficient of variation (COV), i.e. the ratio between the SD and the SUVmean.

CNR was calculated for up to two pathological uptakes per patient. If available, suspected lymph node metastases were chosen first. Otherwise, pathologic uptakes in the prostate or skeleton were selected. A volume of interest (VOI) was drawn over each lesion, covering the whole lesion and some margin, and a spherical local background VOI with a diameter of 1.5 cm was drawn next to the hotspot. CNR was calculated as the difference between SUVpeak in the lesion VOI and the SUVmean in the local background VOI, divided by the SD in the latter. SUVpeak in each lesion VOI was calculated as the average SUV in a 3 × 3 × 3 pixel cube, centred on the SUVmax in the VOI.

### Qualitative analysis

#### Pilot study

To reduce the number of images to be visually evaluated for image quality, a pilot study was performed. Four patients were randomly selected for visual assessment of image quality. This evaluation was performed by two experienced nuclear medicine physicians in consensus. All 84 image series for each patient were viewed and the two to four best images from each acquisition time were selected. This assessment was performed only by visual analysis, not knowing the results from the quantitative analysis. By combining the results from all four patients, the images that were considered best from each acquisition time were chosen for further evaluation (AT of 4 with *β* 1000 and 1100; AT of 6 with *β* 800, 900, and 1000; AT of 8 with *β* 700, 800, and 900), with one image as a reference (AT of 16 with *β* 500). Images with acquisition times of over 2 min/bed position (AT >8) were, after this pilot study, not regarded as relevant for the further analysis aimed at finding the lower limit regarding AT.

#### Assessment of image quality

The eight image series from the pilot study were evaluated for image quality along with the images obtained with AT 16 and *β* 500 (regarded as a reference). Two nuclear medicine physicians individually evaluated the remaining 31 patients in a blinded fashion. The physicians were not aware of the reconstruction parameters or acquisition times used for each patient and were randomly given the nine image series from each patient. The images regarded as being of poor quality were marked, as was the image regarded as having the highest quality. Poor image quality was defined as either the presence of hotspots in the images due to noise, mimicking pathologic uptakes, or pathologic uptakes detected on the reference image (which was correctly identified for all patients by the physicians) but not detected on images with lower AT. The overall image quality was visually assessed, taking the noise level, sharpness, and lesion detectability into account.

### Statistical analysis

Continuous patient parameters are presented as mean ± SD and categorical variables as a percent (%). Noise level, SUVmax, and SUVpeak were tested for normality using the Shapiro-Wilks test. Because the variables were not normally distributed, all quantitative PET data are shown as a median ± interquartile range at the 25th and 75th percentiles.

## Results

### Quantitative assessment

**Fig. 1:**
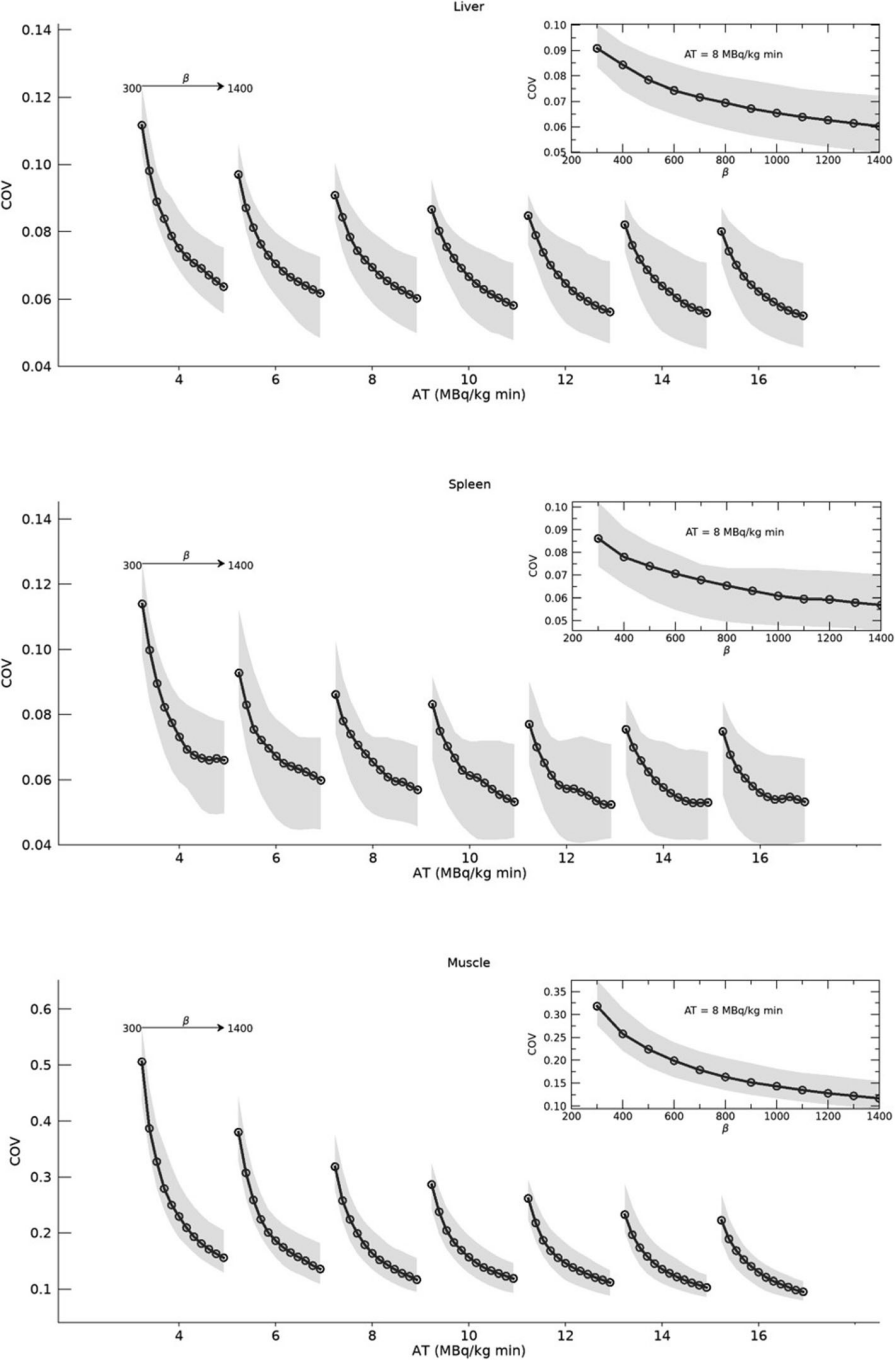
Image noise levels*.* Coefficient of variation (COV) in liver, spleen, and muscle ROIs, as a function of *β* value and activity time product (AT). The solid black line with circles represents the median of all patients (*n* = 33) and the grey shaded area shows the 25th–75th percentile range. The inserts show detailed views for AT = 8 MBq/kg

Figure 1 shows noise levels (COV) in the liver, spleen, and muscle. The COVs in the spleen and liver were of a similar magnitude and showed an essentially equal dependence on *β* and AT. The COV measured in the muscle was higher due to a lower uptake of [^18^F]PSMA-1007 and, therefore, had a stronger dependence on *β* and AT. In general, the noise level had a stronger dependence on *β* than on AT. As expected, image noise was minimized at maximum AT (16 MBq/kg min) and maximum *β* (1400) but there was only a small decrease above AT 10.

**Fig. 2:**
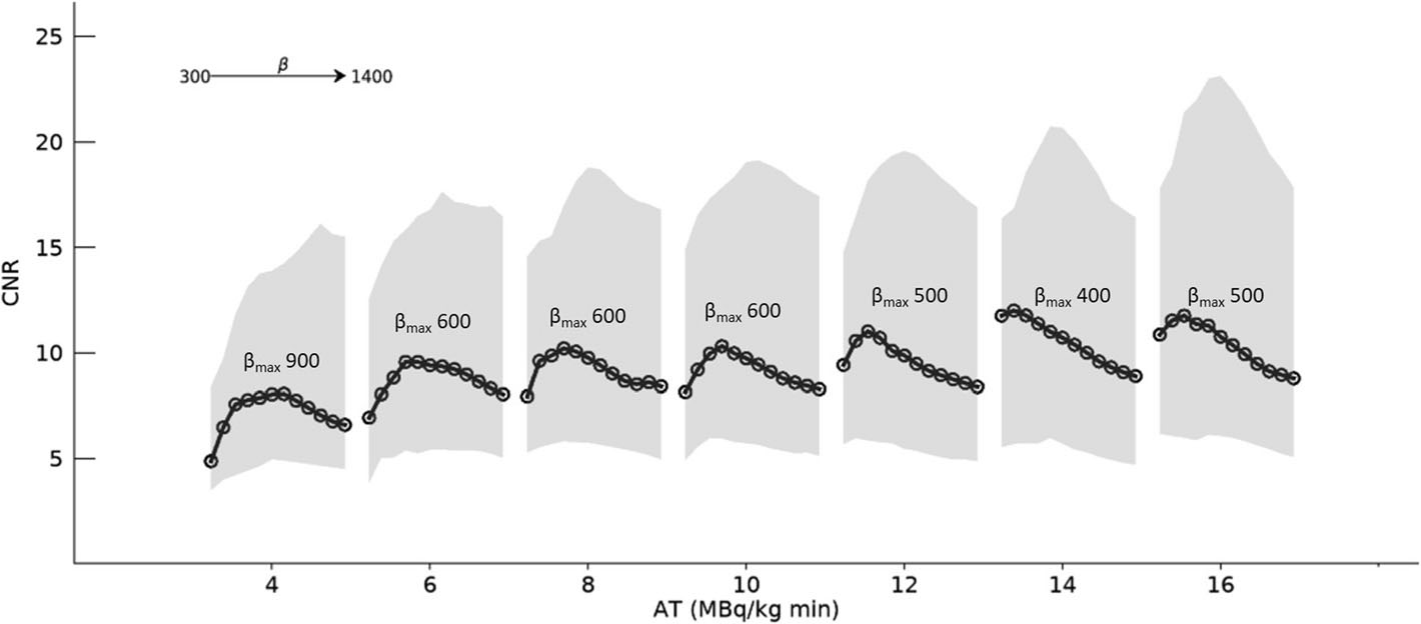
Contrast-to-noise ratio measured in pathological lesions. The median value (solid black line with circles) and 25th–75th percentile ranges (shaded grey area) are shown. The highest CNR for each AT is obtained at *β*_max_, shown in the figure

Figure 2 shows the CNR for all pathological lesions included in the analysis (*n* = 51). The median CNR increased slowly with AT from approximately 6 to 12, was substantially lower at 4, and was higher at AT 14–16. The 25th percentile CNR was relatively constant with increasing AT, implying that there was a subset of pathological lesions with low CNR which did not improve with increasing AT.

The CNR curves for each AT had an optimal *β* value, which gave the highest median CNR. The optimal *β* appears to decrease with increasing AT, from 900 at AT = 4 to 400–500 at AT 12–16.

The median (25th–75th percentile range) SUVmean in the liver, spleen, and muscle was 13.1 (11.3–14.5), 10.6 (8.4–15.0), and 0.4 (0.4–0.5), respectively. As expected, no dependence on either AT or *β* could be noted for SUVmean.

### Qualitative assessment

#### Pilot study

The best image series for each AT is shown in Table 1. In general, the 1-min acquisition time (AT of 4) was regarded as being of too poor image quality. However, two of these image series were selected for further evaluation because we wanted to find the lowest possible AT.

**Table 1:**
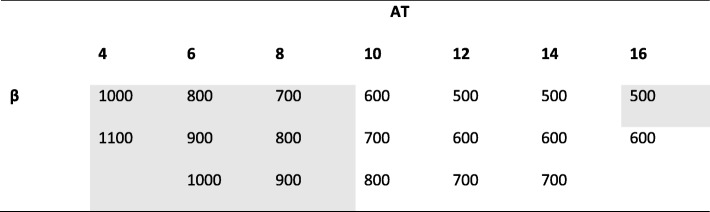
Image quality from the pilot study. The *β* values for the image series that were ranked as the best in terms of overall image quality for each AT based on the pilot study of four patients. The image series highlighted in grey were further evaluated, in a blinded fashion, for image quality. Note that all 84 image series for each patient was visually assessed

#### Assessment of image quality

**Fig. 3:**
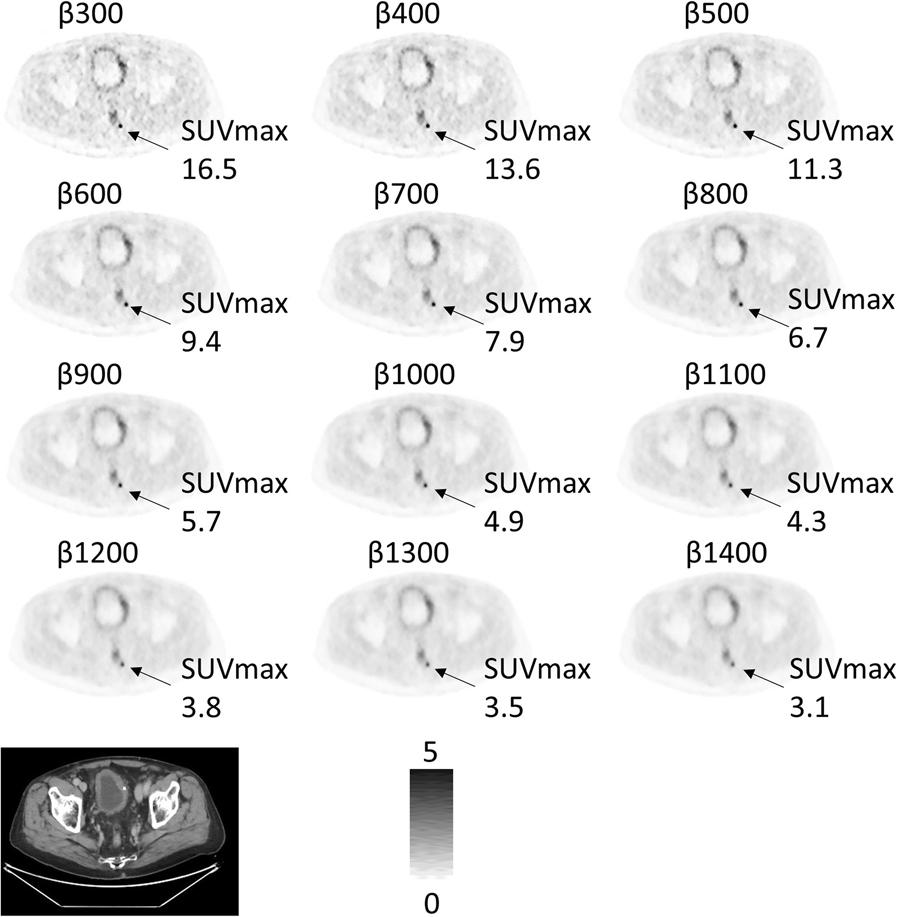
Example of images with different *β* values for AT 8*.* The SUVmax stated for a small perirectal lymph node metastasis

**Fig. 4:**
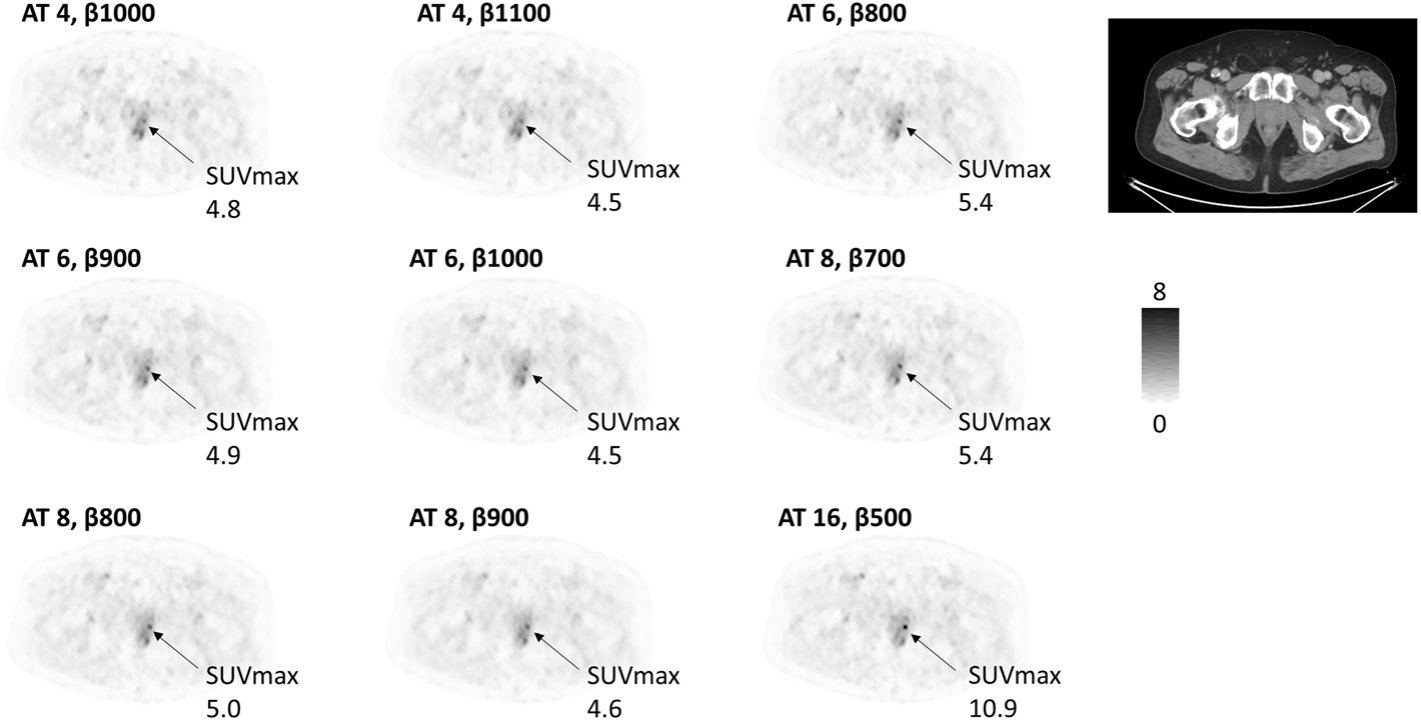
Example of images with different AT and different *β* values. An uptake in the prostate is indicated, and the SUVmax stated

The two observers both ranked all the AT of 16 images as having the best image quality. The largest number of patients with unacceptable image quality was found for AT of 4, then gradually decreasing for AT of 6, AT of 8, and AT of 16 (Table 2). Figure 3 shows a patient example with images of all *β* values (300–1400 with increments of 100) for AT of 8. The SUVmax varied considerably between different *β* values. Figure 4 shows a patient example with selected ATs and *β* values. The PSMA-uptake in the prostate is clearly seen on the AT of 16, *β* 500 image. It is hardly visible on the AT of 4-images. Although the SUVmax does not differ much between the different images shown for AT of 4, 6, and 8, the lesion is more visible for AT of 6 and AT of 8 due to the lower noise level and higher CNR.

**Table 2:** Assessment of image quality. The number of patients with unacceptable image quality in [^18^F]PSMA-1007 PET-CT examinations assessed by two observers

	Observer #1	Observer #2
**AT 4,*β*1000**	23 (74%)	30 (97%)
**AT 4,*β*1100**	23 (74%)	28 (90%)
**AT 6,*β*800**	1 (3%)	4 (13%)
**AT 6,*β*900**	3 (10%)	1 (3%)
**AT 6,*β*1000**	7 (23%)	2 (6%)
**AT 8,*β*700**	0	1 (3%)
**AT 8,*β*800**	0	1 (3%)
**AT 8,*β*900**	0	1 (3%)
**AT 16,*β*500**	0	0

## Discussion

The quantitative image analysis shows that, as expected, longer acquisition time (higher AT) resulted in higher image quality, i.e. lower noise levels and higher contrast-to-noise ratios. In terms of image noise, there was not much improvement from increasing the AT above 10 MBq/kg min (Fig. 1). This holds true even for tissues with low uptake, as shown by data from muscle ROIs. The median COV decreased rapidly with increasing *β*, especially for low acquisition times and low *β* values. As *β* increases, the slope of the COV curve decreases. The inflexion point appears around *β* 500–700, meaning that a change of *β* at a level above this value leads to a smaller difference in the COV than an equal change of *β* at a level below this value. This behaviour is illustrated in the inserts of Fig. 1, where it is seen that (for AT = 8), the COV in, e.g. muscle decreases by 13 % (0.26 → 0.22) for *β* 300 → 400, but only by 5 % (0.135 → 0.128) for *β* 1100 → 1200.

The median CNR for the pathological lesions improved slightly with AT, and some improvement was noted even when AT was increased from levels above 10. On the other hand, the 25th percentile CNR did not improve with AT, indicating that there was a subset of low-uptake lesions whose CNR did not increase with longer acquisition times. The median CNR curves for each value of AT had an optimal *β*, which decreased with an increasing acquisition time. Intuitively, this can be explained as follows: The CNR is decreased by high noise at the lowest *β* values and by a decreased SUVpeak at the highest *β* values. Figure 3 shows a clear example of decreasing lesion SUVmax with increasing *β*, from 16.5 at *β* = 300 to 3.1 at *β* = 1400. At the lowest *β* values, CNR is however decreased because of the high standard deviation in the local background (high noise). Since the noise decreases with increasing *β*, there is an optimum *β* somewhere in between where CNR is maximized. As AT increases, image noise decreases but SUVpeak or SUVmax is less affected. Thus, less noise regularization (lower *β*) is required to reach optimal CNR.

Considering the results of the quantitative analysis alone, it appears that an AT of 10 and a *β* value of approximately 600 is a reasonable choice of parameters.

One should aim for images of high quality but also consider the possibility of either decreasing administered activity or increasing patient throughput through a shorter acquisition time. Our qualitative analysis suggests using an AT of 8, as the number of unacceptable images was low. *β* values of 700–900 appear to perform equally well. In general, there was only a slight difference in the images analysed after the pilot study (Fig. 4). Highly avid tumours were seen regardless of reconstruction parameters but faint uptakes in small anatomical structures (such as lymph nodes or physiologic uptakes in ganglia) were less visible for low ATs.

When one combines the results from the quantitative and qualitative analyses, the use of an AT of 8 with a *β* value of 700 seems reasonable.

We found that the optimal *β* value for different ATs for CNR was lower compared with the preferred *β* value in the visual analysis. This has also been shown in a previous study for [^18^F]fluorocholine [13]. Thus, it seems that CNR alone cannot replace visual analysis by physicians.

Comparison with different *β* values and acquisition times for the BSREM reconstruction for [^68^Ga]Ga-PSMA-11 PET-CT has been investigated before [14, 15]. Ter Voert et al. [15] found that the best *β* value for pelvic [^68^Ga]Ga-PSMA-11 PET-CT was in the range of 400–550 for a 2 min acquisition time and a median injected dose of 128 MBq. Lindstrom et al. [14] found a slight overall preference for a *β* value of 900 after administration of 2 MBq/kg and an acquisition time of 2 min, but also a high interobserver variability. In both studies, the accumulation time was 60 min, as is recommended for [^68^Ga]Ga-PSMA-11 but not for [^18^F]PSMA-1007. Except for the current study, no similar studies for [^18^F]PSMA-1007 PET exist. A recent study [16] compared BSREM with OSEM for [^18^F]PSMA-1007, but only used a single *β* value and acquisition time after an accumulation time of 60 min. That study used a PM-based PET-CT system and a default producer setting for the BSREM with a *β* value of 350 and time-of-flight disabled. This was compared to OSEM using 3 iterations/18 subsets, a 5.5-mm filter and the time-of-flight was enabled. Under these conditions, they found higher SUV and tumour-to-background ratios for BSREM compared to OSEM, especially for small and highly avid lesions. Optimization of BSREM has also been published on other radiopharmaceuticals, which include [^18^F]fluorodeoxyglucose [17–20], [^18^F]fluorocholine [13], and [^18^F]fluciclovine [21], with different optimal *β* values and acquisition times. The differences depend on different bio-kinetics of the radiopharmaceutical, and different amounts of administered activity, which cause different amounts of signal in the images.

These findings should be viewed in light of some limitations. First, only a few patients were analysed in the pilot study and not all image series were individually assessed for image quality. The goal here was to test many reconstructions, therefore, it was not possible to assess all 84 image series per patient. Second, due to the large number of images, only two nuclear medicine physicians were available to interpret the images. It should also be noted that the results obtained in the present study are only valid for 2-h accumulation time and for [^18^F]PSMA-1007.

## Conclusion

An AT of 8 (for example 4 MB/kg with an acquisition time of 2 min) with a *β* value of 700 performs well with respect to noise level, CNR and visual image quality assessments. If the AT is increased, a lower *β* value can be used.

## Data Availability

The datasets used and/or analysed during the current study are available from the corresponding author on reasonable request.

## Abbreviations

AT: Administered activity × acquisition time
BSREM: Block-sequential regularization expectation maximization algorithm
CNR: Contrast-to-noise ratio
COV: Coefficient of variation
OSEM: Ordered subset expectation maximization
PET-CT: Positron emission tomography with computed tomography
PSMA: Prostate-specific membrane antigen
ROI: Region of interest
SiPM: Silicon photomultiplier
SD: Standard deviation
SUV: Standardized uptake value
VOI: Volume of interest
